# Supplementary material for: Complexome profiling of the Chlamydomonas psb28 mutant reveals TEF5 as an early PSII assembly factor
Source: Plant Cell. 2025 Mar 17;37(6):koaf055. doi: 10.1093/plcell/koaf055 (PMC12164590; doi:10.1093/plcell/koaf055)
Supplement: koaf055_Supplementary_Data [file koaf055_supplementary_data.zip › psb28_interactive.zip!/plots/intensity/qualityPlots/peptides/LMR1_Cre09.g393765.t1.1.html]

### Cre09.g393765.t1.1

Trivial name: LMR1  
SeqCoverage [median]: 46.50  
Mass: 22.37 kDa  
  
 

| Sequence | MissedCleavage | Mass [Da] | wtIntensity | muIntensity | Score |
| --- | --- | --- | --- | --- | --- |
| AGETLSEVLSK | 0 | 1132.60 | 3903.28 | 6330.95 | 138.66 |
| DNQVIK | 0 | 715.39 | 267.95 | 0.00 | 107.74 |
| DNQVIKLPIDKFTVR | 2 | 1785.01 | 547.31 | 0.00 | 29.34 |
| EILGGIGTTYR | 0 | 1178.63 | 0.00 | 779.62 | 94.69 |
| GISTDEFLR | 0 | 1036.52 | 3701.16 | 1141.66 | 70.92 |
| KGDTLESIAK | 1 | 1060.58 | 457.72 | 808.92 | 91.66 |
| LSDRDREILGGIGTTYR | 2 | 1921.00 | 1406.57 | 3165.31 | 34.95 |
| SVTVDQILGINPDVKPGK | 1 | 1879.04 | 2467.59 | 9026.04 | 89.98 |
| VVEGQTILLPAQK | 0 | 1394.81 | 7639.25 | 10225.37 | 162.26 |
|  |  |  | 20390.8 | 31477.9 |  |
